# AWT020: a novel fusion protein harnessing PD-1 blockade and selective IL-2 Cis-activation for enhanced anti-tumor immunity and diminished toxicity

**DOI:** 10.3389/fimmu.2025.1537466

**Published:** 2025-02-18

**Authors:** Fan Ye, Jianing Huang, Xiaoli Cheng, Shih Chieh Chen, Fang Huang, Wen-Chin Huang, Botong Hua, Ella Li, Jenny Jiang, Hanna Lin, Matthew Siegel, Eric Liao, Ji Wang, Bella Yue, Wenli Shi, Yanghua Xu, Xin Wang, Jiaming Wang, Yuyuan Yan, Honglin He, Eugene Liu, Binfeng Lu, Ziyang Zhong

**Affiliations:** ^1^ Anwita Biosciences, San Carlos, CA, United States; ^2^ Center for Discovery and Innovation, Hackensack Meridian Health, Nutley, NJ, United States

**Keywords:** PD-1, IL-2 (interleukin-2), tumor infiltrate lymphocyte, CD8, natural kiiler cells

## Abstract

**Background:**

The clinical success of the immune checkpoint inhibitor (ICI) targeting programmed cell death protein 1 (PD-1) has revolutionized cancer treatment. However, the full potential of PD-1 blockade therapy remains unrealized, as response rates are still low across many cancer types. Interleukin-2 (IL-2)-based immunotherapies hold promise, as they can stimulate robust T cell expansion and enhance effector function - activities that could synergize potently with PD-1 blockade. Yet, IL-2 therapies also carry a significant drawback: they can trigger severe systemic toxicities and induce immune suppression by expanding regulatory T cells.

**Methods:**

To overcome the challenges of PD-1 blockade and IL-2 therapies while enhancing safety and efficacy, we have engineered a novel fusion protein, AWT020, combining a humanized anti-PD-1 nanobody and an engineered IL-2 mutein (IL-2c). The IL-2c component of AWT020 has been engineered to exhibit no binding to the IL-2 receptor alpha (IL-2Rα) subunit and attenuated affinity for the IL-2 receptor beta and gamma (IL-2Rβγ) complex, aiming to reduce systemic immune cell activation, thereby mitigating the severe toxicity often associated with IL-2 therapies. The anti-PD-1 antibody portion of AWT020 serves a dual purpose: it precisely delivers the IL-2c payload to tumor-infiltrating T cells while blocking the immune-inhibitory signals mediated by the PD-1 pathway.

**Results:**

AWT020 showed significantly enhanced pSTAT5 signaling in PD-1 expressing cells and promoted the proliferation of activated T cells over natural killer (NK) cells. In preclinical studies using both anti-PD-1-sensitive and -resistant mouse tumor models, the mouse surrogate of AWT020 (mAWT020) demonstrated markedly enhanced anti-tumor efficacy compared to an anti-PD-1 antibody, IL-2, or the combination of an anti-PD-1 antibody and IL-2. In addition, the mAWT020 treatment was well-tolerated, with minimal signs of toxicity. Immune profiling revealed that mAWT020 preferentially expands CD8^+^ T cells within tumors, sparing peripheral T and NK cells. Notably, this selective tumoral T-cell stimulation enables potent tumor-specific T-cell responses, underscoring the molecule’s enhanced efficacy and safety.

**Conclusion:**

The AWT020 fusion protein offers a promising novel immunotherapeutic strategy by integrating PD-1 blockade and IL-2 signaling, conferring enhanced anti-tumor activity with reduced toxicity.

## Introduction

PD-1, a crucial immune checkpoint molecule, suppresses T-cell immunity when engaged with its ligand, Programmed Death-Ligand 1 (PD-L1) (1). Notably, PD-1 expression is significantly increased in tumor-infiltrating T cells, making it an attractive target for promoting T-cell activation in tumor tissues (2, 3). Blockade of the PD-L1/PD-1 axis has become a standard treatment for various cancer indications (4). Although durable responses are seen in a subset of patients with cancer, single-agent anti-PD-1 therapy yields modest response rates in most cancer types (5). Several factors may contribute to the limited clinical response, including a suppressive tumor microenvironment (TME) that impairs T-cell immunity and insufficient numbers of tumor-infiltrating T cells (6, 7). Overcoming these limitations may be achieved by employing combination approaches that deliver more robust T-cell stimulation within the TME.

IL-2 is a critical cytokine that stimulates the proliferation and activation of T cells and NK cells (8, 9). High-dose IL-2 (HD IL-2) has received regulatory approval for the treatment of metastatic melanoma and metastatic renal cell carcinoma, demonstrating an overall response rate of 10-25% in these patient populations. Notably, the responses observed with IL-2 therapy tend to be durable, with some patients experiencing long-lasting tumor control and improved survival outcomes. A recent study showed that HD IL-2 therapy demonstrated antitumor efficacy in melanoma patients who experienced disease progression after ipilimumab treatment (10, 11). In addition, the combination of anti-PD1 and HD IL-2 led to a high response rate with durable response in renal cell carcinoma, suggesting that these two agents could have synergistic activity (12). However, the application of HD IL-2 in the clinic is limited primarily due to severe immune-related adverse events (irAEs) due to a systemic activation of immune cells (13–15). Binding to IL-2Rα may be associated with vascular leak syndrome during HD IL-2 therapy (16). Therefore, several IL-2 variants with abolished IL-2Rα binding (“No-α” IL-2) have been developed to improve the safety and anti-tumor efficacy (17–20). However, these “No-α” IL-2 variants still activate T cells and NK cells systemically and only demonstrate a marginal increase of the maximum tolerated dose in clinical trials compared to wild-type IL-2 (20, 21). Another challenge is the short half-life of IL-2, which requires a frequent treatment schedule (22).

To overcome the limitations of current PD-1 blockade and IL-2 therapies, we have rationally engineered AWT020 - a fusion protein comprised of a humanized PD-1 nanobody and an optimized IL-2 mutein (IL-2c). The overarching goal in developing AWT020 is to synergistically enhance the therapeutic efficacy of both anti-PD-1 antibodies and IL-2-based immunotherapies, while simultaneously improving the safety profile. AWT020 leverages the differential expression of PD-1 between peripheral T cells and tumor-infiltrating lymphocytes (TILs). By selectively binding to PD-1 on TILs, AWT020 delivers a dual benefit: it blocks the inhibitory PD-1 pathway and provides IL-2 signaling to promote the expansion of tumor-antigen-specific T cells. Importantly, the IL-2 mutein component of AWT020 has been engineered to have diminished binding to the IL-2 receptor alpha (IL-2Rα) subunit and attenuated affinity for the beta and gamma subunits (IL-2Rβγ). This design ensures that IL-2 signaling is tightly controlled and dependent on PD-1 binding, which presents the IL-2 mutein to IL-2Rβγ on the cell surface of TILs in a cis configuration within the TME. AWT020 offers a distinctive fusion of PD-1 blockade and IL-2 activation within a single therapeutic agent and is aimed at selectively revitalizing immune responses within the TME while mitigating systemic toxicity associated with uncontrolled immune cell activation.

## Materials and methods

### Engineering and expression of IL-2 fusion proteins

The protein sequences of IL-2 (P60568) and IL-15 (P40933) were obtained from Uniprot. The gene sequences were synthesized by Twist Bioscience with optimized codon for ExpiCHO expression. The binding surface of IL-2 and IL-2 receptors were determined using crystal structure 1z92 and 2erj. Various IL-2 mutein were generated with modified affinity to IL-2 receptors and fused to a humanized anti-HSA nanobody discovered in-house. The leading candidate, IL-2c mutant, contains a peptide derived from human IL-15 to replace amino acid 29-40 in IL-2 (Patent NO. US11897930B2). AWT020 contains two humanized anti-PD-1 nanobodies, a human IgG4 Fc domain, and an IL-2c mutant fused to the C-terminal of Fc with a (G4S)3 linker. The reference molecule, αhPD1-IL-2x, is composed of the same anti-human PD-1 antibody as AWT020 and a more potent IL-2x mutein. IL-2x contains three mutations, F42A, Y45A, and L72G. These three mutations are known to abolish the binding to IL-2Rα but retain the affinity to IL-2Rβγ.

The sequences of two anti-mouse PD1 antibodies, αmPD1 and xmPD1, were obtained from patent US20220401480A1 and patent US20190263877A1, respectively. Two mouse surrogates were created: mAWT020 contains an αmPD1 antibody and an IL-2c mutein, and xmAWT020 contains an xmPD1 antibody and an IL-2c mutein (**Supplementary Table S1**). mAWT020 and xmAWT020 share similar binding affinity to mouse PD1 and identical IL-2c. αmPD1-IL-2x was made with the αmPD1 and an IL-2x mutein. xmPD1-IL-2x was made with xmPD1 antibody and an IL-2x mutein. The genes of different fusion proteins were inserted into pAS-puro vector and transiently transfected in ExpiCHO cells by ExpiFectamine CHO transfection kit (Thermo Fisher Scientific), transfected ExpiCHO cells were cultured in ExpiCHO-S cell culture medium (Thermo Fisher Scientific) for 7-12 days. The culture medium was harvested for protein purification.

### STAT5 phosphorylation assay

Wild-type human T-lymphocyte cell line Hut 78 or PD-1 overexpressing Hut-78 cell line (Hut 78/PD-1) were plated into 96 well deep plates at 0.12E6/15 μL/well with HBSS and treated with 15 μL/well varying concentrations of AWT020 or AWT020Iso or rhIL-2 at 37°C for 40 minutes. A stock solution of AWT020 was formulated at 5 mg/mL in 25 mM Tris-HCl plus 5% sucrose buffer, PH 7.2, and diluted with HBSS. HBSS only was used as the negative medium control.

To block AWT020 binding to PD-1 on Hut 78/PD-1 cells, Hut 78/PD-1 cells were pre-incubated at 37°C for 1 hour with by the parental anti-PD1 antibody, the same anti-PD1 that was used as the fusion partner in AWT020, before treating with AWT020 or AWT020Iso.

pSTAT5 signaling was measured by HTRF technology, according to manual of Phospho-STAT5 (Tyr694) cellular kit. At the end of the treatment period, cells were lysed by adding 10 μL/well supplemented lysis buffer containing blocking reagent and incubated for 30 minutes at room temperature under shaking. After homogenization by pipetting, 16 µL of cell lysate were transferred from the 96 well deep plate to HTRF 96well low volume plate. pSTAT5 signal was captured by adding 4 μL 40-fold detection buffer diluted pSTAT5 Eu Cryptate antibody (donor) and pSTAT5 d2 antibody (acceptor) mixture to each well. Then the plate was cover with a plate sealer and incubated overnight at room temperature.

### Human T cell activation and proliferation assay

Frozen human peripheral blood mononuclear cells (PBMCs) from healthy donors were obtained from STEMCELL Technologies. On the day of assay, PBMCs were thawed and plated at 20,000 cells/well in a 96-well U bottom plate with 100 ul of T cell medium (TCM) containing RPMI-1640, 1% GlutaMAX, 10% fetal bovine serum, 2% human serum, and 100 units/ml penicillin/streptomycin. For pre-activated PBMCs, 1:200 dilution of ImmunoCult™ Human CD3/CD28 T Cell Activator was included in the TCM. The plates were incubated at 37°C and 5% CO_2_ overnight.

The next day, pre-activated and un-activated PBMCs were treated with 100 ul of TCM containing serially diluted AWT020, AWT020iso or αhPD1-IL-2x. PBMCs were incubated at 37°C and 5% CO_2_, and cell culture medium was changed by replacing 100 ul of TCM containing various respective concentrations of AWT020, AWT020iso or αhPD1-IL-2x every 2-3 day. After 7-days of culture for the pre-activated PBMCs, or 10-days of culture for the un-activated PBMCs, cells were collected for staining and flow cytometry analysis.

### Tumor RNA isolation and gene expression profiling

0.5 x 10^6^ B16F10 cells was subcutaneously inoculated into C57/BL6 mice. Following a 7-day period for tumor growth to achieve an average size ranging between 60 to 100 mm^3^, the mice were treated with 1 mg/kg αmPD-1, αmPD1-IL-2x, or mAWT020. On day 13, the mice were sacrificed, and tumors were isolated. Tumors samples were homogenized using a gentleMACS Dissociator (Miltenyi Biotec #130-093-235) using the built-in RNA extraction program. RNA was then isolated using RNeasy Plus Mini Kit (QIAGEN #74134) according to the manufacturer’s protocol and quantified with a Microvolume UV-Vis Spectrophotometer (Thermo Scientific NanoDrop One). RNA expression analysis was conducted using a NanoString nCounter Analysis System (NanoString nCounter Sprint Profiler) with a Mouse PanCancer Immune Profiling Panel comprising 770 genes from diverse immune cell types, common checkpoint inhibitors, cancer/testis antigens, and genes covering both the adaptive and innate immune response. The gene expression analysis was conducted following manufacturer’s instructions. Briefly, tumor RNA was hybridized with a CodeSet containing target-specific biotinylated Capture Probe and color-coded Reporter Probe. The hybridized mixture was then loaded into a cartridge, where excess probes were removed, and the purified hybridized RNA-CodeSet complex was immobilized onto streptavidin coated slides for imaging and counting by the nCounter Analysis System. The obtained results were analyzed using the nSolver Analysis Software.

### Flow cytometry and antibodies

Tumors and whole blood were collected from CT26 tumor-bearing BALB/c mice. Tumor samples were processed into single-cell suspensions described previously. Tumor single-cell suspensions and whole blood were washed with FBS stain buffer (Catalog No. 554656, BD Biosciences) and then incubated with fluorophore-conjugated antibodies of T cell and NK cell markers for 30 minutes at 4°C. Stained tumor and blood cell samples were then washed twice with stain buffer, and result acquisition was performed with an Attune NxT flow cytometer (Invitrogen). FCS file data was analyzed in Attune NxT Software v3.2.1 (Invitrogen). Antibodies employed for flow cytometry are listed here: CD4 (clone GK1.5, Catalog No. 56-0041-82, Invitrogen), CD8 (clone 53-6.7, Catalog No. 45-0081-82, Invitrogen), NKp46 (clone 29A1.4, Catalog No. 25-3351-82, Invitrogen).

### Animals and tumor models

Animal studies were conducted according to the NIH animal care guidelines and following protocols approved by the Institutional Animal Care and Use Committee (IACUC) from Anwita Biosciences. Balb/c and C57BL/6 mice were obtained from the Jackson Laboratory. MC38 murine colon cancer cell line, CT26 murine colon cancer cell line, EMT6 murine mammary carcinoma cell line, and B16F10 murine melanoma cell line were acquired from American Type Culture Collection (ATCC). For subcutaneous tumor models, 0.5-1 x 10^6^ tumor cells were injected subcutaneously into the right flank of the hind leg of Balb/c or C57BL/6 mice. Tumor implanted mice were randomized into different groups with the averaged tumor size of 60 to 100 mm^3^. The mice bearing tumors that were smaller than 40 mm^3^ or larger than 150 mm^3^ were excluded from the randomization. The fusion proteins were administered via intraperitoneal (IP) injection.

### CD8 T cell and NK cell depletion

To deplete CD8 T cells, anti-mouse CD8a antibody (Clone 2.43, from Bio X Cell) was administered intraperitoneally at 15 mg/kg to mice on Days -1, 6, and 13, relative to the day of tumor implantation (Day 0). Similarly, to deplete NK cells, anti-mouse CD161 antibody (NK 1.1, from Bio X Cell) was administered intraperitoneally at 10 mg/kg to mice on Days Days -1, 6, and 13, relative to the day of tumor implantation (Day 0).

## Results

### Molecular design of AWT020

**Figure 1:**
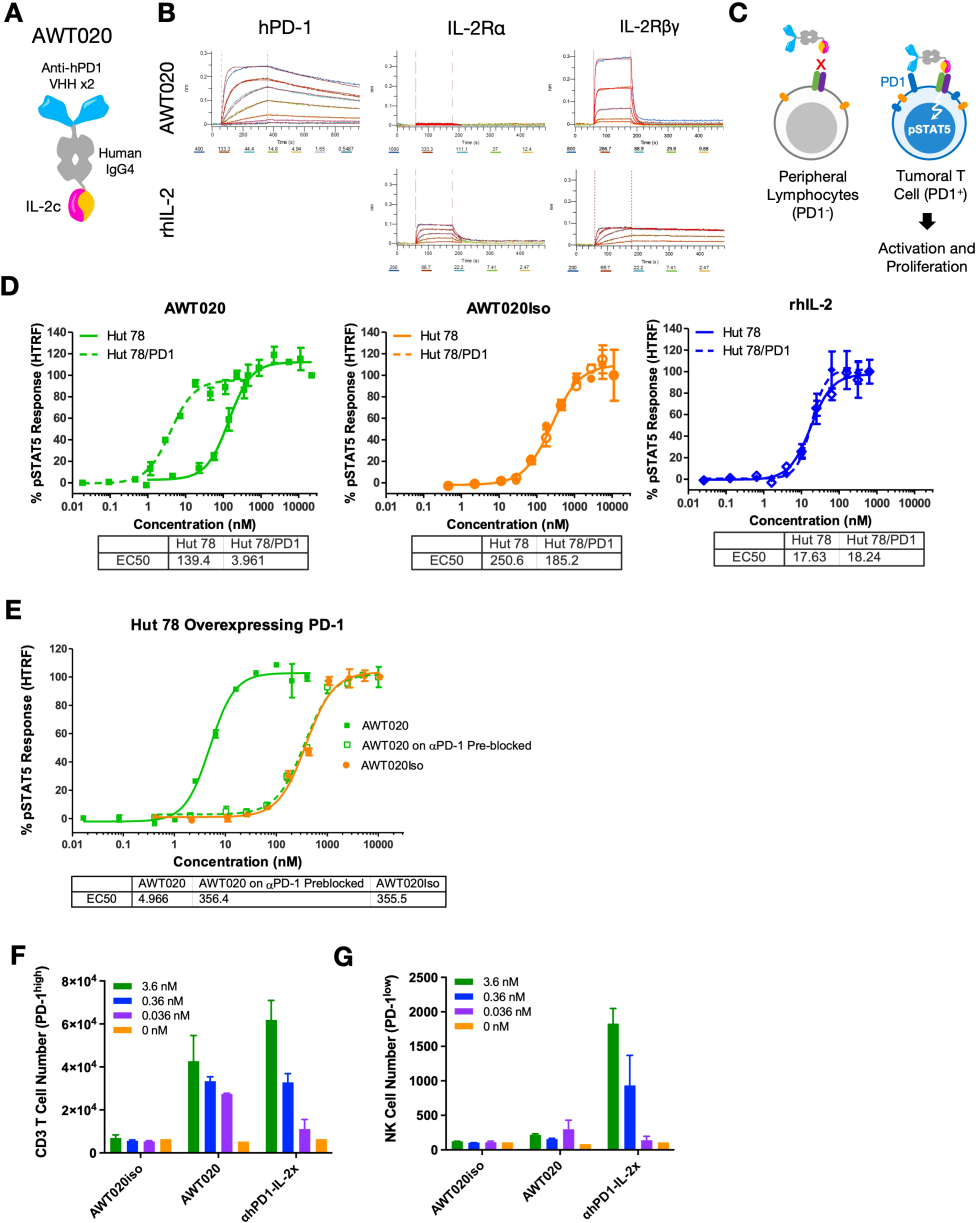
Engineering and characterization of a PD1-IL-2 fusion protein AWT020. **(A)** Schematic representation of the protein conformation of AWT020. Each AWT020 protein comprises two humanized nanobodies that bind to human PD-1, a human IgG4 Fc domain and an IL-2c mutein with reduced affinity to human IL-2Rβγ. **(B)** Octet binding sensorgraphs of AWT020 to human PD-1, human IL-2Rα and human IL-2Rβγ, in comparison to rhIL-2. **(C)** Schematic representation of the proposed mechanism of T cell activation by AWT020. Without PD-1 expression, IL-2c in AWT020 weakly associates to IL-2Rβγ (left); with PD-1 expression, AWT020 anchors on the cell surface and activates IL-2Rβγ in cis configuration (right), inducing the activation and proliferation of target T cell. **(D, E)** STAT5 phosphorylation was measured by an HTRF signal in Hut 78 cells, and Hut 78 cells stably expressed human PD-1 (Hut 78/PD1). **(F)** Flow cytometry analysis of the proliferation of human primary T cell in response to the treatment of various IL-2 fusion proteins. T cells were pre-activated with anti-CD3 and anti-CD28 antibodies to induce PD-1 expression. **(G)** Flow cytometry analysis of the proliferation of human primary NK cells in response to the treatment of various IL-2 fusion proteins.

AWT020 is a bi-functional fusion protein comprised of a humanized nanobody specifically targeting the human PD-1 protein and an engineered human IL-2 mutein (IL-2c) (**Figures 1A, B**). To mitigate potential systemic toxicity related to IL-2, we designed AWT020 with high affinity towards human PD-1 while concurrently reducing its affinity towards IL-2 receptors. By establishing a disparity in affinities, we intended for the high affinity PD-1 binding to govern the cell targeting of AWT020 (**Figures 1A, B**, **Table 1**). The engineered IL-2c mutein in AWT020 resulted in minimal affinity toward IL-2Rα and reduced affinity toward IL-2Rβγ compared to rhIL-2 (**Figure 1B**, **Table 1**). Additionally, an αhPD1-IL-2x fusion was developed, utilizing the identical anti-PD-1 antibody as AWT020 but incorporating a distinct IL-2x mutein. IL-2x mutein contains F42A, Y45A, and L72G mutation to abolish the affinity toward IL-2Rα while retaining the binding affinity to IL-2Rβγ (23). Employing the IL-2x fusion protein as a control enables us to evaluate the necessity of further diminishing binding to IL-2Rβγ to achieve our design’s optimal therapeutic window.

**Table 1:** Dissociation constant (KD, M) of AWT020 to human and cynomolgus monkey IL-2 receptors and PD-1.

		rhIL-2	AWT020
IL-2Rα	Human	2.58 × 10^-8^ ± 4.09 × 10^-10^	N.D.
	Cyno	1.99 × 10^-8^ ± 2.60 × 10^-10^	N.D.
IL-2Rβγ	Human	7.79 × 10^-10^ ± 6.16 × 10^-12^	3.46 × 10^-7^ ± 7.46 × 10^-9^
	Cyno	1.29 × 10^-9^ ± 1.18 × 10^-11^	3.15 × 10^-7^ ± 9.07 × 10^-9^
PD-1	Human	N.D.	5.02 × 10^-9^ ± 1.62 × 10^-11^
	Cyno	N.D.	5.21 × 10^-9^ ± 1.97 × 10^-11^

N.D., Not Detected.

### Induction of pSTAT5 signaling of AWT020 is dependent on PD-1 binding

Given the substantial decrease in binding affinity to IL-2Rβγ, it was anticipated that AWT020 elicited a weak activation of IL-2Rβγ. However, for PD-1^+^ cells, the high affinity PD-1 antibody in AWT020 is expected to anchor itself to the cell surface, facilitating the presentation of IL-2c to IL-2Rβγ in a cis configuration (**Figure 1C**). To investigate the dependency of IL-2 signaling on PD-1 expression, we compared the phosphorylation of STAT5 in Hut78 cells with and without PD-1 expression (**Figure 1D**). AWT020 stimulated STAT5 phosphorylation in Hut 78/PD-1 cells with an EC50 of 4.81 ± 1.95 nM (mean ± SD; n = 3), demonstrating 27-fold higher activity compared to the EC50 of 130 ± 17.7 nM (mean ± SD; n = 3) observed in Hut 78 cells. In contrast, both recombinant human IL-2 (rhIL-2) and the non-PD-1 targeting IL-2c control (AWT020iso) showed no preference for inducing STAT5 phosphorylation in either Hut 78 or Hut 78/PD-1 cells. Further, when PD-1 binding sites on Hut 78/PD-1 cells were blocked through pre-treatment with a saturating amount of parental anti-PD-1 antibody, the potency of pSTAT5 induction by AWT020 was diminished to a level comparable to that elicited by the non-PD-1 targeting AWT020iso (**Figure 1E**), confirming the dependency of IL-2 signaling on the binding of PD-1.

### AWT020 preferentially stimulates the proliferation of PD-1^high^ T cells, but not NK cells

IL-2 is known to induce the proliferation of T cells and NK cells. PD-1 expression was found to be upregulated significantly in activated T cells but not NK cell (2, 3, 24). To assess the ability of AWT020 in proliferating primary immune cells, αCD3 and αCD28 activated human CD3 T cells with high PD-1 expression or human NK cells with low PD-1 expression were treated with AWT020iso, AWT020, or αhPD1-IL-2x. Compared to AWT020iso, both AWT020 and αhPD1-IL-2x treatment significantly increased the number of PD-1^high^ human CD3 T cells (**Figure 1F**). However, only αhPD1-IL-2x induced the proliferation of PD-1^low^ human NK cells (**Figure 1G**). These data suggest that AWT020 has higher selectivity toward PD-1 expressing T cells than NK cells compared to αhPD1-IL-2x.

### mAWT020 demonstrates superior anti-tumor efficacy compared to either the anti-mPD-1 antibody, IL-2 alone, or their combination

**Figure 2:**
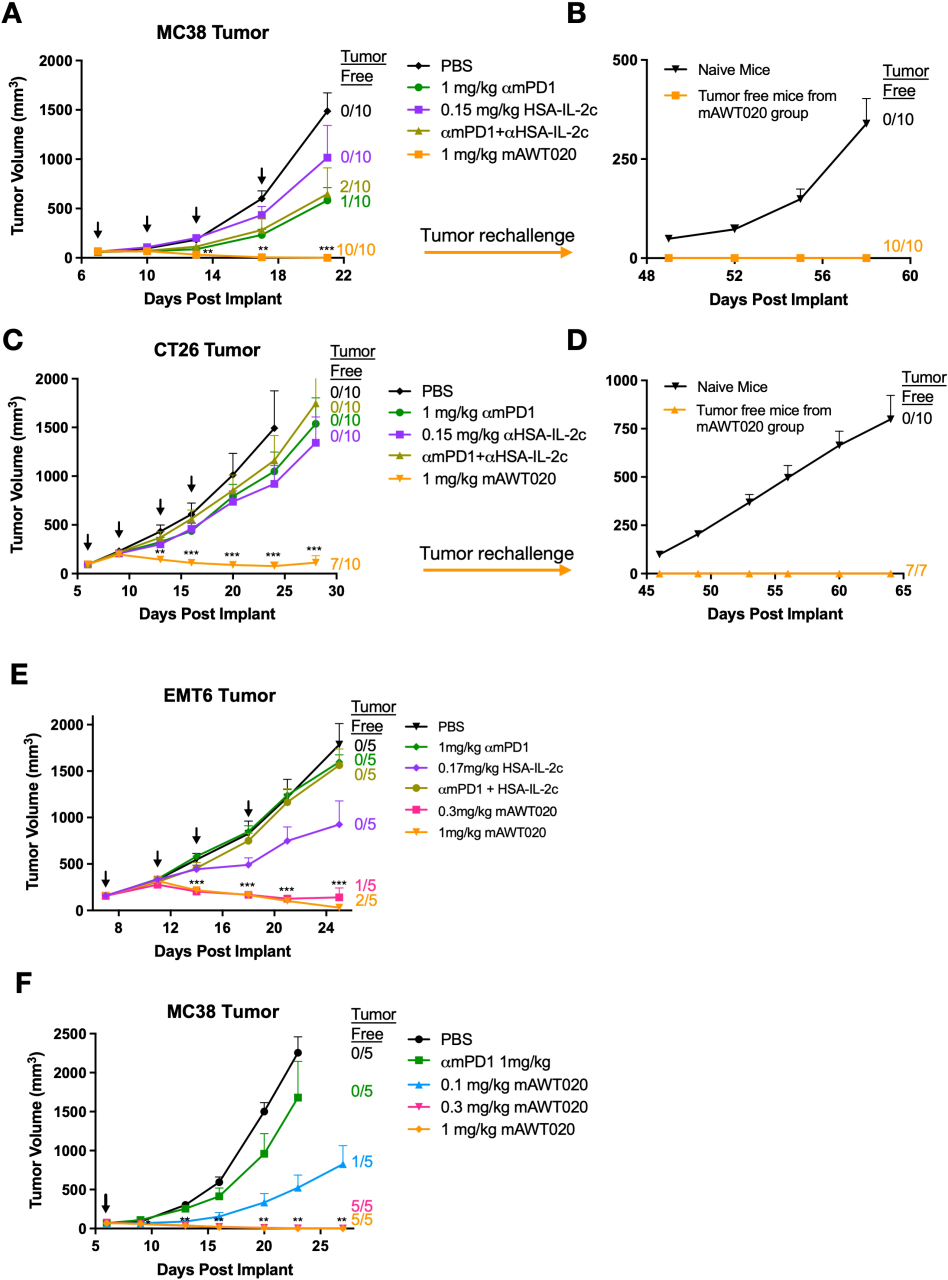
mAWT020 has superior anti-tumor immunity than αmPD1 antibody. Anti-tumor activity of mAWT020 tested in various subcutaneous models including **(A, B, F)** MC38 subcutaneous model, N=10 **(C, D)** CT26 subcutaneous model, N=10, and **(E)** EMT6 subcutaneous model, N=5. Tumor bearing C57/BL6 mice or BALB/c mice were treated with αmPD1, IL-2c, the combination of αmPD1 and IL-2c via IP dosing, with a dose that is molar equivalent to ~ 1 mg/kg of mAWT020. The treatment schedule is indicated in black arrows, Asterisks indicate ANOVA significance values. *P < 0.05; **P < 0.01; ***P < 0.001.

To evaluate the anti-tumor activity of AWT020 in mice, which lacks binding affinity to mouse PD-1, a mouse surrogate named mAWT020 was produced. In the subcutaneous MC38 colon carcinoma model, mice treated with mAWT020 (1 mg/kg, twice-weekly, or biw) demonstrated a significant reduction in tumor size compared to those treated with anti-mPD-1 (αmPD1), half-life extended IL-2c (HSA-IL-2c), or the combination of αmPD1 and HSA-IL-2c. Strikingly, by day 21, all ten mice treated with mAWT020 became tumor-free (**Figure 2A**). Similar results were observed in the subcutaneous CT26 colon carcinoma model. Mice treated with mAWT020 (1 mg/kg, biw) exhibited a great tumor size reduction compared to αmPD1, HSA-IL-2c, or the combination treatment of αmPD1 and HSA-IL-2c. By day 28, 7 out of 10 mice treated with mAWT020 became tumor-free (**Figure 2C**). The tumor-free mice from both the MC38 and CT26 studies were further subjected to a tumor rechallenge study, where they remained tumor-free without any additional treatment (**Figures 2B, D**). These results indicate that mAWT020 treatment induced a strong antitumor effect and long-lasting immunological memory. In the EMT6 breast cancer model (**Figure 2E**), where PD-1 therapy has limited efficacy, both 0.3 mg/kg and 1 mg/kg mAWT020 treatment demonstrated significantly better suppression of tumor growth than αmPD1 or the combination therapy of αmPD1 and HSA-IL-2c. To ascertain the minimum effective dose of mAWT020 necessary for suppressing tumor growth, various dosing levels ranging from 0.1 to 1 mg/kg were evaluated in the MC38 model. The 0.1 mg/kg mAWT020 treatment showed superior tumor growth suppression compared to the 1 mg/kg αmPD1 treatment. Treatment with mAWT020 at doses of 0.3 mg/kg and 1 mg/kg resulted in complete tumor regression in 100% of mice (5 out of 5 animals per group) by days 27 and 24, respectively (**Figure 2F**). Collectively, these results provide compelling evidence that mAWT020 exhibits superior anti-tumor efficacy compared to αmPD1 antibody, HSA-IL-2c administered alone, or their combination.

### mAWT020 specifically promotes the expansion of tumor-infiltrating T cells but not NK cells

**Figure 3:**
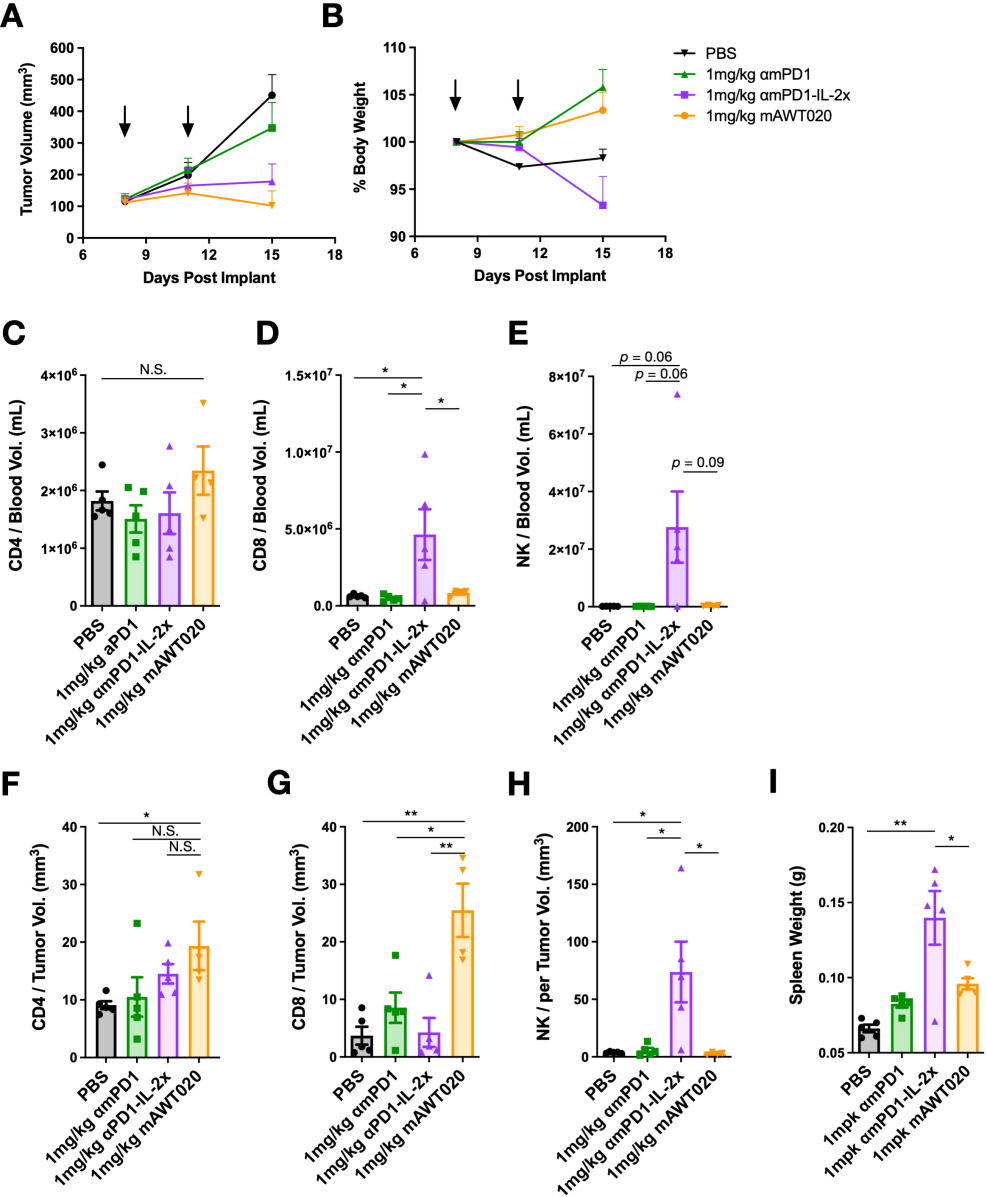
mAWT020 specifically expands tumor infiltrated T cell but not NK cell. **(A)** Tumor growth curve and **(B)** the percentage of body weight change of CT26 tumor bearing mice treated with 1 mg/kg of αmPD1, αmPD1-IL-2x or mAWT020 via IP dosing, N=5. The treatment schedule is indicated in black arrows. At day 15, blood and tumor were collected for FACS analysis of immune cell subtypes **(C-H)**. **(I)** Spleen weight of the CT26 tumor bearing mice treated with 1 mg/kg of αmPD1, αmPD1-IL-2x or mAWT020, N=5. Asterisks indicate ANOVA significance values. N.S. not significant; *P < 0.05; **P < 0.01.

To investigate the mechanism underlying the improved anti-tumor efficacy of mAWT020 compared to αmPD1, CT26 tumor-bearing mice were treated with 1 mg/kg of αmPD1, 1 mg/kg of αmPD1-IL-2x, or 1 mg/kg of mAWT020 (biw) to compare the immune subset profile in blood and tumor. After 2^nd^ dose, both the αmPD1-IL-2x and mAWT020-treated groups exhibited superior suppression of tumor growth compared to the αmPD1-treated group. However, only the αmPD1-IL-2x-treated group displayed reduced physical activity and body weight loss (**Figures 3A, B**). Moreover, the αmPD1-IL-2x-treated group showed significantly enlarged spleens compared to the PBS-treated group, indicating systemic activation of the peripheral immune system. In contrast, the mAWT020 treatment only induced a mild increase in spleen weight. (**Figure 3I**). In the blood, αmPD1-IL-2x treatment resulted in an increase in circulating CD8^+^ T cells and NK cells, whereas mAWT020-treated mice showed no significant change in the total number of T cells or NK cells (**Figures 3C–E**). Interestingly, within the tumor, mAWT020 significantly increased the number of CD8^+^ T cells compared to the PBS or αmPD1-treated groups, but it did not affect NK cell numbers (**Figures 3F–H**). In contrast, αmPD1-IL-2x treatment increased the number of NK cells much more than T cells within the tumor (**Figures 3F–H**). These findings collectively suggest that the activity of mAWT020 is highly specific to tumor-infiltrating T cells. In contrast, treatment with the αmPD1-IL-2x fusion protein exhibited poor tumor and T cell selectivity, as it induced proliferation of immune cells in both the peripheral blood and the tumor tissues.

To conduct comprehensive whole tumor transcriptomic studies, we administered 1 mg/kg of αmPD-1, αmPD1-IL-2x, or mAWT020 biw to C57/BL6 mice bearing B16F10 tumors. On day 13, five out of eight mice per group were sacrificed for gene expression profiling, while the remaining three mice in each group were retained to monitor tumor growth and changes in body weight. (**Supplementary Figures S1A, B**). Both αmPD1-IL-2x and mAWT020 showed better tumor growth suppression than αmPD-1 treated mice. The key genes related to T cell activation and expansion were tested using NanoString nCounter hybridization method. Both αmPD1-IL-2x and mAWT020 treated mice showed higher levels of CD8a and CD8b1 gene expression in tumors compared to PBS or αmPD1 treated mice (**Supplementary Figure S2C**). Moreover, the genes related to T cell activation, such as Pdcd1, Cxcr6, Gzma, Gzmb, and Prf1, also upregulated in the tumor of αmPD1-IL-2x and mAWT020 treated mice. These results support the observation of the elevated CD8 T cell number in tumors, indicating that αmPD1-IL-2x and mAWT020 are capable of both proliferating and activating CD8^+^ T cells in tumors.

### CD8^+^ T cells drive the efficacy of mPD1-IL-2 fusion protein, while NK cells mediate its toxicity

**Figure 4:**
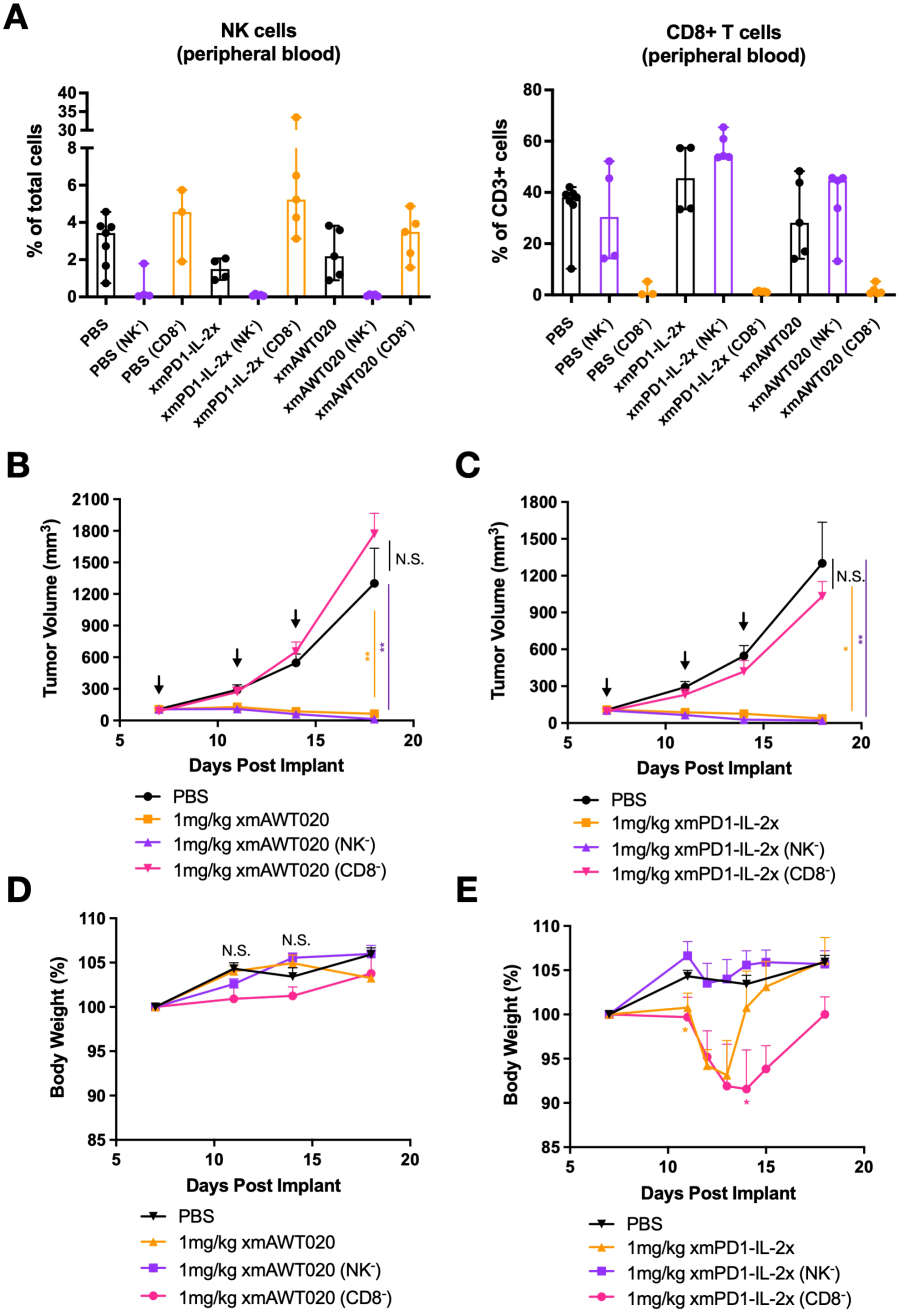
CD8 T cells drive the efficacy of mPD1-IL-2 fusion protein, while NK cells drive its toxicity. **(A)** Percentage of T cell and NK cell in PMBC collected at day 19 from PBS, αmPD1-IL-2x, and mAWT020 treated mice with NK cell (NK^-^) or CD8 T cell (CD8^-^) depletion. **(B, C)** Anti-tumor activity of mAWT020 **(B)** or αmPD1-IL-2x **(C)** in MC38 tumor bearing mice with or without the depletion of NK cell (NK^-^) or CD8 T cell (CD8^-^), N=5. **(D, E)** Percentage of body weight change of mAWT020 treated mice or mPD1-IL-2x treated mice with or without the depletion of NK cell (NK^-^) or CD8 T cell (CD8^-^). Asterisks indicate ANOVA significance values. *P < 0.05; **P < 0.01.

Previous studies have demonstrated that PD-1 therapy acts by activating T cells in the tumor, whereas “No-α” IL-2 therapy promotes the proliferation of both T cells and NK cells (20). To determine which immune cell subtypes drive the anti-tumor activity and toxicity of αmPD1-IL-2x or mAWT020, CD8 T cell or NK cell depletion was performed in MC38 tumor-bearing C57/BL6 mice. The percentage of NK and T cells in the blood were quantified to verify the effect of depletion antibodies (**Figure 4A**). Both mAWT020 and αmPD1-IL-2x were highly efficacious in suppressing MC38 tumor growth compared to the PBS-treated group. However, when CD8 T cells were depleted using an anti-CD8 antibody, both mAWT020 and αmPD1-IL-2x completely lost their anti-tumor effects (**Figures 4B, C**). In contrast, NK cell depletion did not affect the anti-tumor activity of mAWT020 and αmPD1-IL-2x, as tumor growth suppression was similar to that observed in the treatment group without NK cell depletion (**Figures 4B, C**). These results indicate that the mechanism underlying the anti-tumor activity of PD1-IL-2 fusion protein is primarily driven by CD8 T cells rather than NK cells. As previously observed, treatment with 1 mg/kg of mAWT020 in C57/BL6 mice showed no signs of toxicity or body weight loss (**Figure 4D**). However, treatment with 1 mg/kg of αmPD1-IL-2x induced approximately 8% body weight loss after the second dose (**Figure 4E**). Interestingly, depletion of CD8 T cells had a minor effect on body weight change, while depletion of NK cells prevented body weight loss in the αmPD1-IL-2x-treated group (**Figure 4E**). These data suggest that the systemic proliferation or activation of NK cells in αmPD1-IL-2x-treated mice is the cause of toxicity.

To determine the maximum tolerated dose of mAWT020 and whether high-dose mAWT020 treatment induces proliferation of NK cells or T cells in the periphery, naive BALB/c and C57/BL6 mice were treated with 1 mg/kg αmPD-1, 1 mg/kg αmPD1-IL-2x, or mAWT020 at doses of 1 mg/kg, 3 mg/kg, and 10 mg/kg. Consistent with previous observations, treatment with 1 mg/kg of αmPD1-IL-2x resulted in body weight loss after the second dose in both BALB/c and C57/BL6 mice (**Supplementary Figure S2A**). In contrast, mAWT020 treatment groups showed no body weight loss at any tested doses in both BALB/c and C57/BL6 mice (**Supplementary Figure S2A**). The immune cell profile in the blood was also examined. In BALB/c mice, treatment with 1 mg/kg of αmPD1-IL-2x resulted in a 3.01-fold increase in CD3^+^ T cells and a 187.27-fold increase in NK cells, while there were no significant changes in T cell and NK cell numbers in the mAWT020-treated groups at all doses (**Supplementary Figures S2B, C**). In C57/BL6 mice, treatment with 1 mg/kg of αmPD1-IL-2x induced a 20.06-fold increase in T cells and a 27.58-fold increase in NK cells, whereas mAWT020 treatment had no significant effect on the number of T cells and NK cells in the peripheral blood at any doses (**Supplementary Figures S2B, C**). These data indicate mAWT020 has strong antitumor efficacy and a favorable toxicity profile.

### Toxicokinetics of AWT020 in cynomolgus monkey

The cynomolgus monkey was a suitable species for toxicology assessment based on the ability of AWT020 to bind to both its PD-1 and IL-2Rβγ targets in monkeys with similar activity as in humans (**Table 1**). In a non-GLP single-dose tolerability study, a total of 4 cynomolgus monkeys (two male and two female) were assigned to 5 mg/kg or 10 mg/kg treatment groups, respectively. The pharmacokinetics of AWT020 in non-GLP study is shown in **Table 2**.

**Table 2:** Mean toxicokinetics of AWT020 in cynomolgus monkey following single intravenous infusion administration.

Dose (mg/kg)	Sex	No.	t_1/2_(h)	C_max_ (μg/mL)	AUC_0-t_ (h·μg/mL)	V_z_ (mL/kg)	Cl (mL/h/kg)	MRT_0-t_ (h)
5	Male	1	30.3	170	5,870	36.9	0.842	47.2
	Female	1	131	72.9	4,930	159	0.842	93.8
10	Male	1	84.7	173	9,200	125	1.02	80.4
	Female	1	54.9	136	7,110	99.4	1.26	53.3

AUC_0-t_, area under the concentration-time curve from time zero to last measurable concentration; C_max_, maximum serum concentration; h, hour(s); t_½_, half-life of elimination; Vz, volume of distribution at terminal phase; Cl, clearance; MRT_0-t_, mean residence time from time zero to last measurable concentration.

**Figure 5:**
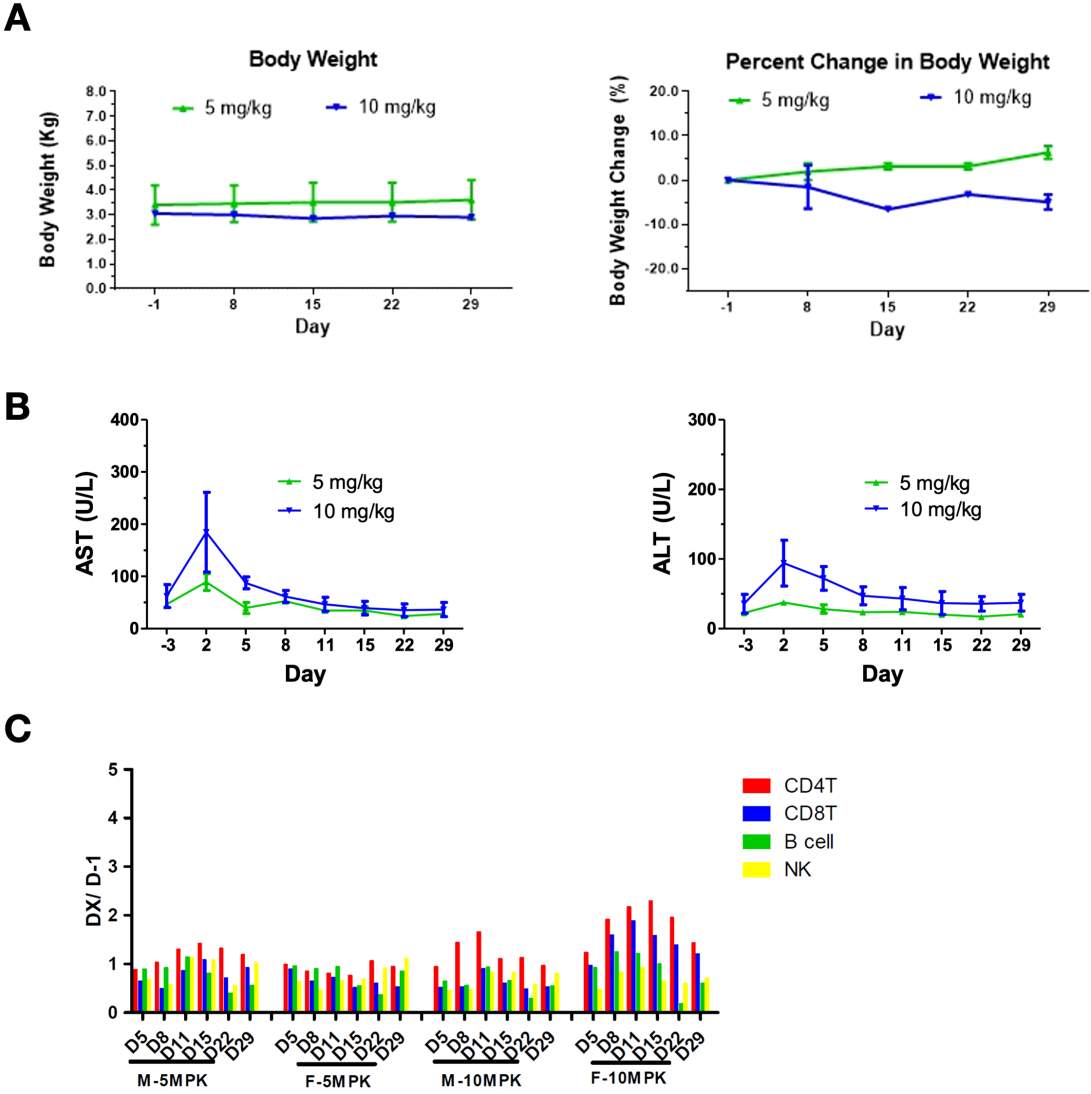
Safety profile of AWT020 in Cynomolgus Monkey. **(A)** Body weight change in Kg (left) or percentage (right) of cynomolgus monkey treated with 5 mg/kg or 10 mg/kg AWT020 single dose. **(B)** The change of AST (left) and ALT (right) of cynomolgus monkey treated with 5 mg/kg or 10 mg/kg AWT020 single dose. **(C)** Lymphocyte count after a single dose of AWT020 at 5 mg/kg (5MPK) or 10 mg/kg (10MPK) in male monkey (M) or female monkey (F).

No mortality or moribundity was noted in any animals at any interval of data collection during the study. The changes in mean body weight over time and percent change in body weight relative to predose weight over time are shown in **Figure 5A**. The 10 mg/kg group showed an approximately 7% decrease in body weights over the study duration from Day 1 to 29 (**Figure 5A**). Minor changes in blood chemistry and hematology were noted. At 10 mg/kg, aspartate transferase (AST) increased from 62.0 ± 31 U/L at predose to 184.5 ± 108 U/L on Day 2 and alanine aminotransferase (ALT) elevated from 35.5 ± 19 U/Lat predose to 94.0 ± 47 U/L on Day 2, and completely recovered to baseline levels by Day 15 (**Figure 5B**). C-reactive protein (CRP) increased from 7.9 ± 5.2 at predose to 25.3 ± 12.1 mg/L at 24 hours post dose at 5 mg/kg and increased from 4.9 ± 0.7 at predose to 18.8 ± 4.0 mg/L at 12 hours post dose at 10 mg/kg. Changes in CRP returned to the baseline level by Day 15. Further, an increase in CD4, CD8, B and NK cell counts of less than 2-fold on Day 8 was noted at 10 mg/kg and returned to the baseline levels by Day 15 (**Figure 5C**). No increase in the levels of serum IFN-γ, IL-1β, IL-5, IL-10, IL-6, TNF-α, and MCP-1 were observed in any of the treated animals. In conclusion, the high dose of 10 mg/kg was well tolerated after a single administration to cynomolgus monkeys via intravenous infusions.

## Discussion

In the present study, we designed and evaluated AWT020, a bi-functional fusion protein composed of a humanized nanobody targeting the human PD-1 protein and an engineered human IL-2 mutein (IL-2c). Our aim was to mitigate potential systemic toxicity associated with IL-2 by designing AWT020 with high affinity towards human PD-1 while concurrently reducing its affinity for IL-2 receptors. Our preclinical studies in both anti-PD-1-sensitive and anti-PD-1-resistant mouse tumor models demonstrated that mAWT020 exhibited superior anti-tumor efficacy compared to either anti-PD-1 antibody or IL-2 alone, or their combination. The efficacy of mAWT020 was primarily driven by the expansion and activation of CD8^+^ T cells within the tumor microenvironment, while NK cell activation contributed minimally to tumor control and primarily resulted in increased toxicity. Interestingly, mAWT020 not only selectively expanded and activated CD8+ T cells in “hot” tumor models (e.g., MC38 and CT26), but also demonstrated the ability to significantly elevate T-cell markers and activation signatures in the “cold” tumor model B16F10. This indicates that mAWT020 has the potential to convert immunologically “cold” tumors into “hot” tumors by enhancing T-cell infiltration and activation in the tumor microenvironment. These findings highlight the importance of targeting PD-1+ T cells in achieving a strong anti-tumor response and underline the therapeutic potential of AWT020 in providing a favorable safety profile while greatly enhancing anti-tumor efficacy.

IL2 receptor complexes are widely expressed by both tissue and peripheral immune cells, including T cells, NK cells, ILCs, and myeloid cells. The pleiotropy of IL-2 limits its efficacy and is a major factor contributing to its toxicity. Despite wild-type IL-2 (Aldesleukin) being the first immunotherapy approved by the FDA, its clinical application is hampered by severe immune-related adverse events (irAEs) and the requirement for a frequent dosing schedule (600,000 - 720,000 Units/kg, q8h, up to a maximum of 14-15 doses, equivalent to about 9,600,000 IU doses, or ~0.037 mg/kg). A targeted approach is necessary to reduce the pleiotropy of IL-2 and enhance its therapeutic efficacy while minimizing toxicity. It is thought that the toxicity is mediated by binding to IL2Rα and the efficacy is also limited by IL2Rα expression on Treg cells. To enhance the safety and efficacy of wild-type IL-2, various “No-α” IL-2 variants that do not bind to IL2Rα and possess extended half-lives have been developed. However, the No-α IL-2 muteins still exhibit significant toxicity. For example, Sanofi’s THOR-707 demonstrated a recommended phase 2 dose (RP2D) ranging from 0.024 mg/kg Q3W to 0.032 mg/kg Q3W (21).

PD1 is primarily expressed on tumor antigen-engaged T cells in tumor tissues. Upon activation of T cells by antigens, TCR signaling induces the expression of PD-1 in T cells (25, 26). This mechanism leads to highly specific upregulation of PD-1 on tumor-infiltrated T cells, while circulating T cells express minimal levels of PD-1 (2, 3). PD-1 therapy utilizing monoclonal antibodies to disrupt the PD1-PD-L1 interaction primarily targets tumor-infiltrating T cells and has much less effect on circulating lymphocytes. The good safety profile of anti-PD1 antibodies can be partly attributed to the restricted expression of PD1 on tumor-infiltrating T cells. The anti-PD-1-IL2 fusion protein is then conceived to increase the binding of IL2 mutein to PD1^+^ TILs, thereby enabling low toxicities due to reduced IL2 binding to its receptors in peripheral immune cells while boosting PD1^+^ tumor antigen-specific T cells in the TME.

In recent years, several IL-2 or IL-15 fusion proteins have been developed. As a pioneer in IL-2 fusion proteins, Roche developed CEA-IL-2v and FAP-IL-2v, as well as PD1-IL-2v (23, 27, 28). The IL-2v mutein used in these three fusion proteins is identical—a “No-α” IL-2 containing F42A, Y45A, and L72G mutations. Despite completely different targeting strategies, where CEA-IL-2v and FAP-IL-2v target tumors and PD1-IL-2v targets tumor-infiltrated T cells, these IL-2v fusion proteins showed comparable tolerability in humans, with maximal tolerated doses of approximately 0.3-0.5 mg/kg. These findings suggest that the off-target activity of IL-2v may be the limiting factor for the safety of these fusion proteins. Although “No-α” IL-2 abolished the binding to IL-2Rα, it still binds to IL-2Rβγ with high affinity. Therefore, the distribution of a “No-α” IL-2 fusion protein is determined by the targeting antibody and IL-2Rβγ binding.

Our study, along with others, indicated that the activation of peripheral NK cells can drive the toxicity of PD-1-IL-2 and PD-1-IL-15 fusion proteins (24, 29). NK cells express high levels of IL-2Rβγ but lack IL-2Rα, making them susceptible to off-target toxicity from no-alpha IL-2 and IL-15. Chen et al. developed an anti-PD-1-IL-15 fusion protein with no binding to IL-2Rα or IL-15Rα while maintaining a relatively high affinity to IL-2Rβγ (KD = 12.29 nM) (30). This anti-PD-1-IL-15 fusion protein can activate NK cytotoxicity at 1 nM concentration *in vitro* and induce NK cell proliferation in cynomolgus monkeys starting from 0.2 mg/kg. Xu et al. engineered an anti-PD-1-IL-15 mutein incorporating E46G and V49R mutations to eliminate IL-15Rα binding, along with N1G, E64Q, and D30N mutations to further reduce IL-2Rβ binding affinity (KD > 1500 nM) (24). This anti-PD-1-IL-15 mutein showed better tolerability in mouse models; however, at 5 mg/kg, PD-1-IL-15m induced more than a 30-fold expansion of NK cells in blood and significant body weight loss in mice. This body weight loss could be rescued by NK cell depletion using NK1.1 antibody, indicating NK-dependent toxicity. Shen et al. created an anti-PD-1-IL-15-IL-15Rα fusion protein by fusing IL-15Rα to the C-terminus of IL-15; this molecular format abolished the binding of IL-15 to cell surface IL-15R and significantly reduced IL-2Rβγ binding likely due to steric hindrance (29). In this study, Shen et al. demonstrated that NK is more sensitive to IL-15 than T cells due to its higher expression of IL-2Rβ. *In vitro*, splenocyte proliferation assay showed that IL-15 fusion protein induced NK expansion at 0.3 µg/mL, while IL-15-IL-15Ra fusion protein did not induce NK expansion up to 5 µg/ml. In mouse models, the anti-PD-1-IL-15-IL-15Rα was dosed up to approximately 1.5 mg/kg without showing apparent body weight loss. However, without higher doses, it is impossible to evaluate the tolerability improvement of this molecule. These findings suggest a reduction of IL-2Rβγ binding is required for anti-PD-1-IL-2 or anti-PD-1-IL-15 fusion protein to mitigate NK-mediated toxicity.

Our study demonstrated that fine-tuning the reduction of binding affinity to the IL-2Rβγ complex significantly improved the tolerability of AWT020. The reference molecule used in this study, the anti-mPD1-IL-2x fusion protein, binds to IL-2Rβγ with an affinity comparable to wt IL-2 (~1 nM). In contrast, mAWT020 has a much lower affinity for IL-2Rβγ (>500 nM). The high affinity binding to IL-2Rβγ in αmPD1-IL-2x likely leads to significantly higher NK cell proliferation in the blood, contributing to its limited tolerability with a maximum tolerated dose of 1 mg/kg in mice. In tumors, αmPD1-IL-2x also expands significantly more NK cells than CD8+ T cells. We hypothesize that the overproliferated NK population in anti-mPD1-IL-2x-treated mice may have suppressive effects on tumoral CD8+ T cells due to potential mechanisms such as direct cytotoxicity toward activated CD8+ T cells (31, 32), inhibition of dendritic cells (33, 34), and/or acting as a receptor sink to compete for gamma chain cytokines such as IL-2, IL-15, or IL-21. In contrast, due to the low level of PD-1 expression in NK cells and reduced IL-2Rβγ binding affinity, AWT020, and its mouse surrogate, mAWT020, induce minimal activation or expansion of the NK cells in both human and mouse PBMCs. Consequently, mAWT020 was well tolerated at 10 mg/kg in mice, and AWT020 was well tolerated at 10 mg/kg in the monkeys. The preclinical toxicity profile establishes AWT020 as a best-in-class molecule, delivering robust antitumor efficacy while optimizing tolerability.

Overall, the molecular design of AWT020, with its tailored affinity for PD-1 and reduced binding to IL-2 receptors, represents a promising strategy to enhance the specificity and safety of IL-2-based therapies. The improved tolerability of AWT020 facilitates the complete blockade of the PD1-PD-L1 interaction, thereby preventing the inhibitory signal mediated by PD-1. Concurrently, IL-2R is activated to enhance the activation and proliferation of T cells within the TME. Our study provides compelling evidence supporting the continued development and potential clinical application of AWT020 for cancer immunotherapy. AWT020 is currently being evaluated in a phase 1 clinical trial (ClinicalTrials.gov Identifier: NCT06092580).

## Data Availability

The original contributions presented in the study are included in the article/**Supplementary Material**. Further inquiries can be directed to the corresponding authors.
