# Supplementary material for: AWT020: a novel fusion protein harnessing PD-1 blockade and selective IL-2 Cis-activation for enhanced anti-tumor immunity and diminished toxicity
Source: Front Immunol. 2025 Feb 18;16:1537466. doi: 10.3389/fimmu.2025.1537466 (PMC11880808; doi:10.3389/fimmu.2025.1537466)
Supplement: Supplementary file 1 [file Table1.docx]

**AWT020: A Novel Fusion Protein Harnessing PD-1 Blockade and Selective IL-2 Cis-activation for Enhanced Anti-Tumor Immunity and Diminished Toxicity**

Fan Ye, Jianing Huang, Xiaoli Cheng, Shih Chieh Chen, Fang Huang, Wen-Chin Huang, Botong Hua, Ella Li, Jenny Jiang, Hanna Lin, Matthew Siegel, Eric Liao, Ji Wang, Bella Yue, Wenli Shi, Yanghua Xu, Xin Wang, Jiaming Wang, Yuyuan Yan, Honglin He, Eugene Liu, Binfeng Lu, Ziyang Zhong

**Supplemental Material**

**Supplemental Materials and Methods**

**PD-1 antibody discovery**

The anti-human PD-1 nanobody was discovered in-house. Two llamas were immunized with human PD-1 protein (from Sino Biologics). After the 2nd boost, peripheral blood mononuclear cells (PBMCs) were isolated for the construction of a phage display library. The phage library was panned against human and cynomolgus PD-1 to identify clones with cross-reactivity. High-affinity clones were enriched through additional rounds of panning. The affinity of phage clones was determined by enzyme-linked immunosorbent assay (ELISA). After 3 rounds of panning, high-affinity phage clones were sequenced to determine the antibody sequence. The DNA sequence of anti-PD-1 nanobodies was synthesized and fused to the human IgG4 Fc domain for ExpiCHO expression. The purified anti-PD-1 nanobodies were tested for their function in blocking PD-1-PD-L1 interaction. Leading clones were humanized by modifying the frameworks of llama nanobodies to match the most similar human antibody sequences while retaining the sequence of CDRs.

**Bio-Layer Interferometry (BLI) assay**

The binding activity between PD-1 to PD-1 antibodies, IL-2 to IL-2 receptors were determined using Octet RED96 BLI Label-Free Detection System (Sartorius, Inc.). To determine the binding affinity of PD-1, Anti-Human-Fc (AHC) biosensors were used to capture PD-1 antibody or PD-1 antibody fusion proteins. The coated biosensors were associated with 3-fold serially diluted His-tagged human PD-1 protein (ACROBiosystems, Inc.). For the detection of IL-2Rα or IL-2Rβγ binding, Anti-Penta-HIS (HIS1K) biosensors or Streptavidin (SA) biosensor was used to capture His- or Avitag labeled IL-2 receptors (ACROBiosystems, Inc.) and associated with serially diluted IL-2 or IL-2 fusion proteins. All the test articles, control articles, receptor proteins, and human PD-1 were diluted in 10× Kinetic Buffer Casein (10× KBC) containing 1× phosphate-buffered saline (PBS), 0.02% Tween 20, and 0.1% casein at pH 7.4. Data Analysis HT 10.0.3.7 software was used to obtain the affinity KD values. Bindings from reference samples which contained 10× KBC only without the test articles, or from the reference biosensors without coating with the receptor proteins, were considered as non-specific background. After subtracting the background, specific binding curves were analyzed using the 1:1 binding model. The global fitting was used to fit the serial concentration within the same analyte. The full R2 of the fitting results are all greater than 0.95.

**Isolation of murine tumor lymphocytes**

Tumor samples were harvested from mouse syngeneic tumor models, and surrounding fat and fibrous tissues were removed from each tumor. For better processing cell dissociation, tumor sample was cut into small pieces of 1-3 mm, and then transferred the sample into the gentleMACS C Tube (Catalog No. 130-093-237, Miltenyi Biotec) containing the dissociation enzyme mix (2.35 mL of serum-free RPMI 1640, 100 µL of Enzyme D, 10 µL of Enzyme R, and 12.5 µL of Enzyme A, Catalog No. 130-096-730, Miltenyi Biotec). The gentle MACS C tube was placed on the gentle MACS Octo Dissociator with Heaters (Catalog No. 130-096-427, Miltenyi Biotec), and the selected tumor dissociation program 37C_m_TDK_1 was run. Once completed, the cell pellets were resuspended and transferred to a 30 µm MACS SmartStrainer (Catalog No. 130-110-915, Miltenyi Biotec) placed on a 15 mL tube. An additional 10 mL serum-free RPMI 1640 was used to wash and rinse the residual cells through the MACS SmartStrainer. The cells were then pelleted by centrifugation at 500xg for 5 minutes, and the cell pellets were resuspended in RPMI 1640 with 10% FBS; the cells were ready for further applications, including FACS cell phenotyping and in vitro cell assays.

**Flow Cytometry Analysis**

Blood and tumor-derived cells were labeled with anti-mCD3 (FITC), anti-mCD4 (PE), anti-mCD8 (PerCP-Cy5.5), and anti-mNKP46 (APC). Singlet events were gated based on forward scatter (FSC) and side scatter (SSC). Within the monocyte/lymphocyte population, T cells were defined as mCD3⁺; CD4 T cells as mCD3⁺ mCD4⁺; CD8 T cells as mCD3⁺ mCD8⁺; and NK cells as mCD3⁻ mNKP46⁺. Fluorescence-minus-one (FMO) controls were prepared for each marker to accurately define positive populations, and compensation was performed for both panels to correct for spectral overlap.

**Supplemental Figure legends**

**Figure S1. mAWT020 preferentially activates tumor infiltrated lymphocytes**

**A** B16F10 tumor growth curve in mice treated with 1 mg/kg αmPD-1, αmPD1-IL-2x, or mAWT020. **B** Body weight change in percentage of B16F10 tumor bearing mice treated with 1 mg/kg αmPD-1, αmPD1-IL-2x, or mAWT020. **C-D** mRNA expression profile of immune cells collected from B16F10 tumor or the spleens of B16F10 tumor bearing mice.

**Figure S2.** **mAWT020 is better tolerated in both BALB/c and C57/BL6 mice than mPD1-IL-2x**

**A** Percentage of body weight change in BALB/c (left) or C57/BL6 (right) mice treated with 1 mg/kg αmPD-1, 1 mg/kg αmPD1-IL-2x, or mAWT020 at doses of 1 mg/kg, 3 mg/kg, and 10 mg/kg, biw. **B** Numbers of CD3 T cell in blood. **C** Numbers of NK cells in blood

**Table S1 Comparison of test articles used in the manuscript**

|  | **IL2 variant** | **hPD1 binding** | **mPD1 binding** |
| --- | --- | --- | --- |
| AWT020 | IL-2c (no alpha, IL-2Rβγ attnuated) | Yes | No |
| AWT020iso | IL-2c (no alpha, IL-2Rβγ attnuated) | No | No |
| αhPD1-IL-2x | IL-2x (no alpha) | Yes | No |
| mAWT020 | IL-2c (no alpha, IL-2Rβγ attnuated) | No | Yes (G4C2 clone from US20220401480A1) |
| xmAWT020 | IL-2c (no alpha, IL-2Rβγ attnuated) | No | Yes (xmPD1 from US20190263877A1) |
| αmPD1-IL-2x | IL-2x (no alpha) | No | Yes (G4C2 clone from US20220401480A1) |
| xmPD1-IL-2x | IL-2x (no alpha) | No | Yes (xmPD1 from US20190263877A1) |
