# Supplementary figures and images for: AWT020: a novel fusion protein harnessing PD-1 blockade and selective IL-2 Cis-activation for enhanced anti-tumor immunity and diminished toxicity
Source: Front Immunol. 2025 Feb 18;16:1537466. doi: 10.3389/fimmu.2025.1537466 (PMC11880808; doi:10.3389/fimmu.2025.1537466)

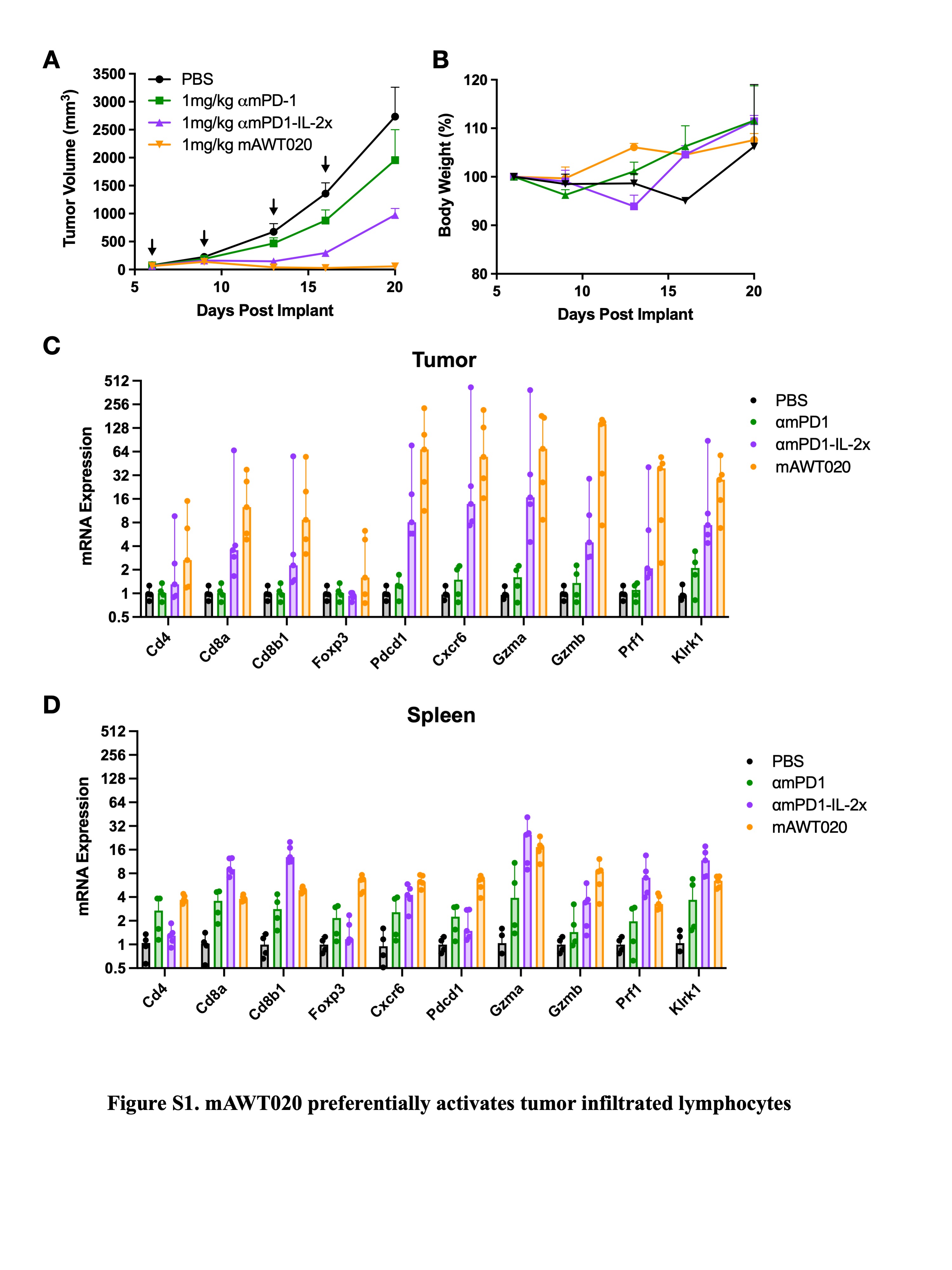

Supplement: Supplementary file 2 [file Image1.jpeg]

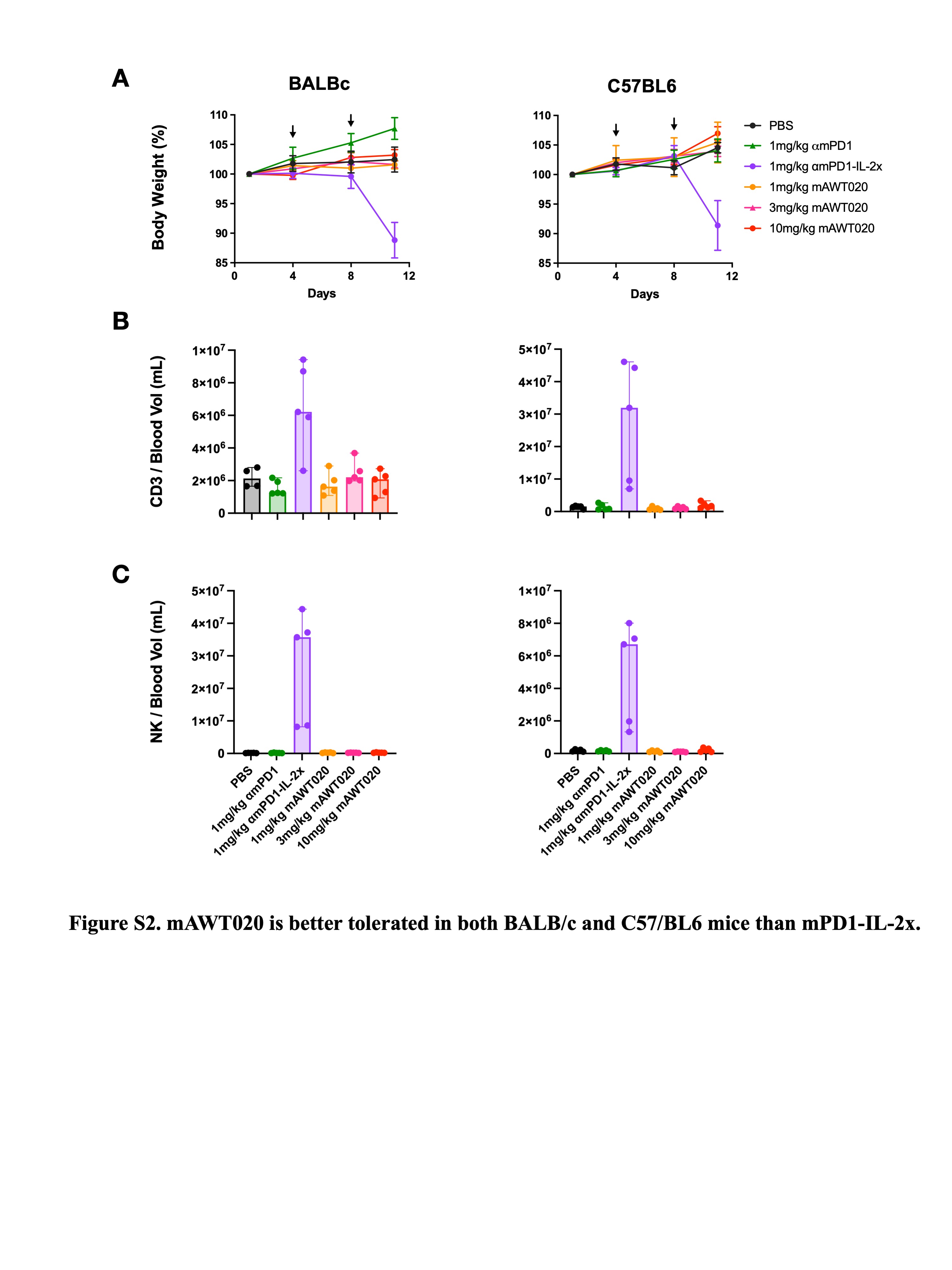

Supplement: Supplementary file 3 [file Image2.jpeg]
